# Visualising the strain distribution in suspended two-dimensional materials under local deformation

**DOI:** 10.1038/srep28485

**Published:** 2016-06-27

**Authors:** Kenan Elibol, Bernhard C. Bayer, Stefan Hummel, Jani Kotakoski, Giacomo Argentero, Jannik C. Meyer

**Affiliations:** 1 Faculty of Physics, University of Vienna, Boltzmanngasse 5, A-1090 Vienna, Austria

## Abstract

We demonstrate the use of combined simultaneous atomic force microscopy (AFM) and laterally resolved Raman spectroscopy to study the strain distribution around highly localised deformations in suspended two-dimensional materials. Using the AFM tip as a nanoindentation probe, we induce localised strain in suspended few-layer graphene, which we adopt as a two-dimensional membrane model system. Concurrently, we visualise the strain distribution under and around the AFM tip *in situ* using hyperspectral Raman mapping via the strain-dependent frequency shifts of the few-layer graphene’s G and 2D Raman bands. Thereby we show how the contact of the nm-sized scanning probe tip results in a two-dimensional strain field with μm dimensions in the suspended membrane. Our combined AFM/Raman approach thus adds to the critically required instrumental toolbox towards nanoscale strain engineering of two-dimensional materials.

Suspended two-dimensional materials such as graphene, MoS_2_ or MoTe_2_ have a wide application profile ranging from ultra-fast electronics and nano-electro-mechanical systems^1^,^2^,^3^,^4^ to ultimately thin functional membranes for chemical species separation^5^,^6^. Characterization of free-standing, atomically thin two-dimensional membranes often employs scanning probe microscopy (SPM) techniques such as atomic force microscopy (AFM) or scanning tunneling microscopy (STM)^7^,^8^,^9^,^10^. Recent work has however highlighted that SPM techniques do not leave the atomically thin membrane mechanically undisturbed during measurements^11^,^12^,^13^,^14^,^15^,^16^,^17^. Instead, SPM measurements lead to local membrane deformations at the location of the scanning tip. Such deformations and the resulting localised strain distribution in the two-dimensional material can complicate SPM measurement interpretation and also lead to degradation in the two-dimensional material upon SPM measurements. On the other hand, in most two-dimensional materials the application of strain leads to changes in opto-electronic properties, which allows strain engineering of the material properties^12^,^18^,^19^,^20^,^21^,^22^,^23^,^24^,^25^. This opens a way to probe such strain-dependent opto-electronic properties based on the application of localised strain through SPM-based techniques^26^,^27^,^28^,^29^,^30^. The effect of strain has however typically only been assessed for two-dimensional materials subjected to either macroscopically applied^31^,^32^,^33^,^34^,^35^,^36^,^37^,^38^ or irreversible strains (via support on corrugated substrates)^25^,^34^,^39^,^40^,^41^,^42^,^43^,^44^, while the extent and distribution of *reversible and localised* strain, as from highly localised SPM techniques, in *suspended* two-dimensional materials remains largely unknown.

Here, through simultaneous AFM and laterally resolved Raman spectroscopy measurements (Raman mapping), we visualise the strain distribution in a freely suspended two-dimensional material membrane from highly localised deformations, adopting few-layer graphene (FLG) as a model system. We use the AFM tip as a nanoindentation probe to controllably induce localised strain in the suspended FLG in the elastic regime, which we visualise using hyperspectral Raman mapping *in situ* (i.e., with the local force from the AFM tip applied) via the strain-dependent frequency shifts of the FLG’s G and 2D Raman bands^31^,^32^,^33^,^34^,^35^,^36^,^37^,^38^,^43^,^45^. In contrast to earlier works, we obtain a laterally resolved map of the non-homogeneous strain distribution in the FLG around and under the AFM tip, whereby we clearly resolve that the nm-sized SPM tip contact results in a two-dimensional strain field with μm dimensions. Our combined AFM/Raman approach thus facilitates visualisation of localised and reversible strain and deformation in two-dimensional materials and thereby enhances the available toolbox towards strain engineering in two-dimensional materials on the nanoscale.

## Results and Discussion

**Figure 1:**
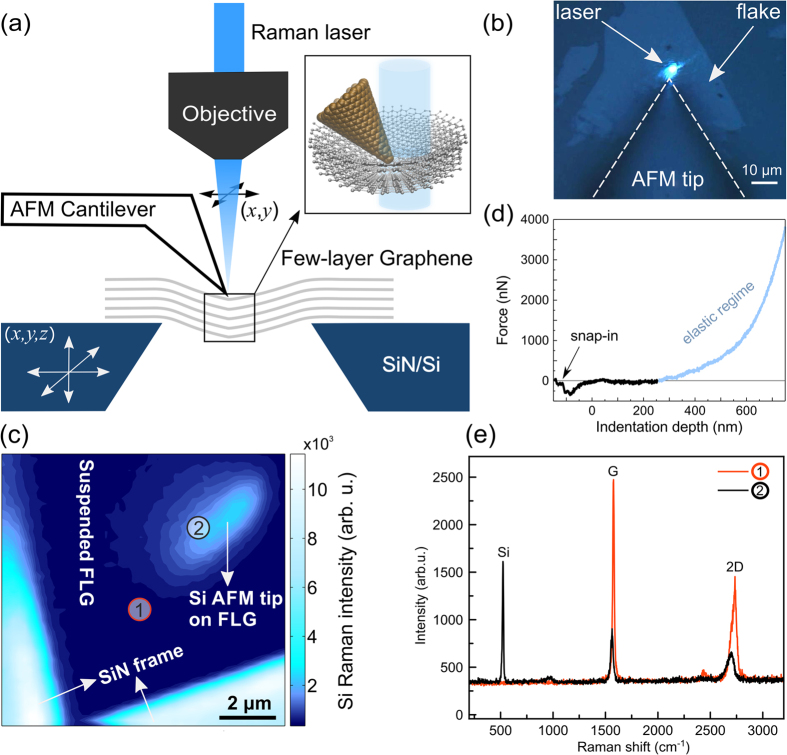
(**a**) Schematic illustration of the AFM-Raman spectroscopy setup. (**b**) An optical microscopy image of a two-dimensional material flake with AFM tip in place (viewed from top). (**c**) A Raman map of Si peak intensity. (**d**) Force-indentation curve measured on the suspended FLG. The origin is defined as the return to zero force after the initial tip snap-in. (**e**) Full Raman spectra for locations far (“1”) from and close (“2”) to the AFM probe, with the locations also marked in the Si intensity map in (**c**).

Figure 1(a) shows a schematic presentation of our simultaneous AFM and hyperspectral Raman mapping experiments on suspended two-dimensional membranes. To prepare the samples of suspended FLG over the single 12 μm × 12 μm holes in holey SiN-covered Si chips (SiMPore), we first exfoliate highly oriented pyrolytic graphite (HOPG) onto SiO_2_ covered Si wafers and then use N-Methyl-2-pyrrolidone (NMP) dissolvable plastic transmission electron microscopy (TEM) grids (Quantifoil) as intermediate carriers to deterministically transfer appropriately sized FLG flakes onto the holey SiN/Si chips. See ref. 14 for further details on the transfer process. The thickness of the FLG is identified from the optical contrast difference on the initial SiO_2_/Si substrate^46^ and then cross-checked by Raman spectroscopy after transfer^47^,^48^. The here presented data was acquired for a FLG flake of 5 layers, with a Raman 2D peak shape consistent with AB Bernal stacking^47^,^48^. Supplementary Figure S1(a) shows a tapping mode AFM image of the membrane, which reveals intrinsic wrinkling in our FLG flake on a lateral μm scale with wrinkle heights of 50 to 250 nm, as well as a small amount of polymeric residues from the intermediate plastic TEM carrier grid used for FLG placement^14^,^49^.

A NT-MDT NTEGRA Spectra coupled AFM-Raman spectrometer is used in our experiments, where the AFM uses a fixed AFM probe (i.e., cantilever with tip) combined with a moveable piezo tube scanner onto which the sample is loaded. The piezo tube allows movement of the sample laterally and vertically under the AFM tip. The laser spot for Raman spectroscopy is independently moveable laterally on the sample surface via a mirror mounted onto a second piezo tube in the optical beam path of the confocal Raman spectrometer (laser wavelength 473 nm; numerical aperture of objective lens of 0.7, resulting in an estimated diffraction limited lateral resolution^50^ of ~400 nm compared to a measured optical laser spot diameter of ~1 μm; measured laser power on sample 3.5 mW; reflected signal from sample fed back into spectrometer with a 600 g/mm grating). This setup thus allows to perform completely independent AFM measurements and hyperspectral Raman mapping on the same sample region. To avoid shadowing of the region of interest next to the AFM tip by the AFM probe cantilever we use optical access AFM probes made of silicon coated with Pt, where the Pt-coated Si tip emanates *in front* of the cantilever at an angle of ~128° (Fig. 1(b), NT-MDT VIT_P_Pt, nominal tip radius 10 nm, spring constant 37.8 N/m measured by thermal noise method^51^, see Supplementary Figure S2 for scanning electron microscopy (SEM) images of the AFM probes).

We estimate the local strain distribution under and adjacent to the AFM tip via the strain-dependent frequency shifts of the FLG’s G and 2D Raman bands^31^,^32^,^33^,^34^,^35^,^36^,^37^,^38^,^43^,^45^, as discussed below. For our AFM-based nanoindentation and simultaneous Raman mapping the AFM was operated in contact mode. During the lateral scanning of the Raman laser spot, the AFM tip was kept on a fixed spot on the surface of the suspended FLG. Figure 1(c) shows a Raman map plotting the intensity of the first order Si signal at 520 cm^−1^ 52, clearly delineating the edges of the hole in the SiN/Si chip over which the FLG is suspended as well as the position of the Si AFM tip on the FLG.

To obtain the force-dependence of the Raman maps (and thus strain), the force applied on the FLG was systematically altered via controlling the cantilever deflection set-point (0.2 nA, 0.6 nA, 1.0 nA for a sum photodetector signal of ~27 nA) of the AFM feedback loop (where Raman maps of unstrained suspended FLG were measured with the AFM probe removed). To quantify the force applied on the membrane, measurements of cantilever deflection versus vertical sample piezo movement were obtained on the FLG membrane, as well as on the rigid SiN/Si chips, and recalculated to force-indentation curves (Fig. 1(d))^53^,^54^. The force-indentation curve of the FLG shows non-linear characteristics, confirming that the FLG is elastically deformed upon AFM-based nanoindentation^7^. When the AFM tip approaches, first a snap-in of the tip is detected where the tip is bent downwards and the FLG presumably curved slightly upwards^14^,^16^. This is followed by a rather flat region in the force-indentation curve (up to ~250 nm indentation depth) where the FLG membrane accommodates the initial tip movement predominantly via the flatting of the initially present 50–250 nm high ripples and wrinkles in the suspended FLG (Supplementary Figure S1(a)), as previously reported^14^,^16^,^55^. Then the FLG membrane enters the elastic deformation regime^56^, in which C-C bond stretching becomes important and which is the focus of our current work. Fitting the force-indentation data for indentation values >250 nm (i.e., for indentation values above which the intrinsic wrinkles have flattened out and the FLG entered the elastic regime) with the model from ref. 7 yields a Young’s modulus in the region of ~0.5 TPa, consistent with previous reports (Supplementary Figure S1(b))^57^,^58^. Importantly, we note that the elastic force-indentation characteristics are reversible (Supplementary Figure S1(b)) and no rupture events (i.e., no irreversible sudden stepped decrease in force upon loading^7^) are observed for the FLG membrane. Consistently, no signs of degradation (e.g. hole formation, rupture etc.) are observed by optical microscopy of the FLG after nanoindentation measurements. This confirms that the FLG membrane is not macroscopically damaged during our measurements.

**Figure 2:**
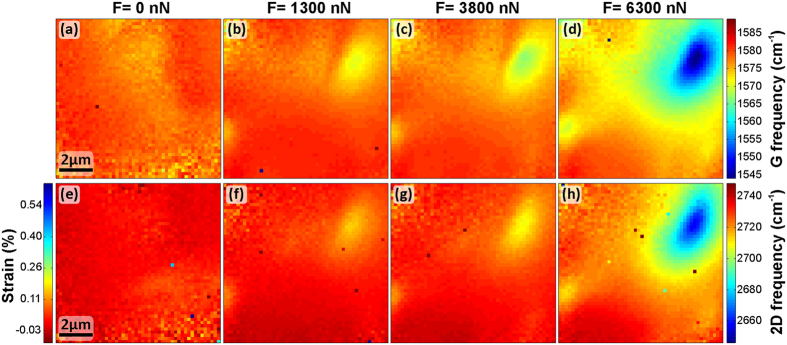
Raman maps of G and 2D frequencies over the entire sampled area (incl. free-standing FLG and FLG supported on SiN/Si; c.f. Fig. 1(c)). Forces applied by AFM probe are (**a,e**) 0 nN (no tip in place), (**b,f**) ~1300 nN, (**c,g**) ~3800 nN, and (**d,h**) ~6300 nN, respectively. Additionally a scale bar to (**e–h**) for estimated strain is given, following ref. 36.

Figure 2 shows the central observations of our nanoindentation study: In Fig. 2(a–d) Raman maps of the G band frequency (*ω*_*G*_) for four different forces applied by the AFM tip (0 nN, i.e., no AFM tip used, ~1300 nN, ~3800 nN, and ~6300 nN) are shown. In Fig. 2(e–h) the corresponding maps of the 2D frequency (*ω*_2*D*_) are presented. The plotted Raman frequencies were obtained by fitting the G and 2D regions of the hyperspectral Raman maps’ individual spectra (examples in Fig. 1(e)) to single Lorentzians. We note that upon removal of the AFM tip after measurements, the Raman signature of the FLG reverts to the initial signature, confirming that the deformations induced by the AFM tip in this study are reversible, i.e., the FLG is only elastically deformed (see also Supplementary Figure S3). We emphasize here, that in particular for the ~1300 nN to ~3800 nN case, the set-points of the contact mode AFM feedback (and thus forces applied by the AFM tip) are within the region of typical settings for AFM contact mode imaging and nanoindentation/force-distance measurements.

Both G and 2D Raman bands in graphitic materials are highly sensitive to the application of strain, where a downshift in frequency of G and 2D peaks indicates tensile strain^31^,^32^,^33^,^34^,^35^,^36^,^37^,^38^,^43^,^45^. Qualitatively our measurements in Fig. 2 show that upon the application of the highly localised force from the AFM tip, with increasing force a localised pattern of downshifts in both G and 2D peak positions evolves, which is centred under the location of the AFM tip on the FLG. For the non-loaded 0 nN control case (Fig. 2(a,e)), we find that the Raman band frequencies of the FLG area supported on the SiN/Si chip are not significantly shifted with respect to these of the suspended FLG area. Assuming that the SiN/Si supported FLG is on a macroscopic scale largely unstrained^59^, this indicates that the suspended FLG area is also macroscopically not significantly strained when not contacted by the AFM tip. When loading the FLG by the AFM tip at an initial force of ~1300 nN, we find in Fig. 2(b,f) that a small, roughly circular region with a small downshift in G (maximum downshift of −10 cm^−1^) and 2D (maximum downshift of −24 cm^−1^) evolves right under the position of the AFM tip (c.f. the Si intensity map in Fig. 1(c)). At the initial indentation load of ~1300 nN the diameter of the downshifted region is ~2 μm. When increasing the force applied to ~3800 nN this region of downshifted G and 2D frequency increases in diameter to ~4 μm (Fig. 2(c,g)). With the maximum applied force in our experiments (∼6300 nN), the region of downshifted G and 2D increases both in the magnitude of maximum downshifts (G to −38 cm^−1^ and 2D to −79 cm^−1^) and also in spatial extent to a diameter of ~7 μm (Fig. 2(d,h)). Since the magnitude of G and 2D downshift is directly related to tensile strain, our measurements directly visualise how far the strain from the contacting AFM tip is spread.

The Raman maps in Fig. 2 further indicate that for ~1300 nN and ~3800 nN between the SiN/Si frame and the AFM tip a several μm-wide region exists which does not show significant G and 2D frequency shifts. This implies that for ~1300 nN and ~3800 nN the deformation induced by the AFM tip is fully accommodated within the central suspended area of the FLG and does not pervade across the entire FLG flake. In other words, the localised application of force by the AFM tip results in a localised strain distribution fully within the suspended FLG, surrounded by a suspended region that remains essentially unstrained. In contrast, for the ~6300 nN case, a gradual shift towards lower G and 2D frequencies is observed over almost the entire measured FLG region, incl. on the SiN/Si support, with only the exception near the SiN/Si corner in the lower left. Thus for the higher load of ~6300 nN the significantly strained region of the FLG extends onto the SiN/Si frame.

To assess the spatial extent of our Raman features in Fig. 2 with respect to the AFM tip geometry, we estimate the maximum possible projected contact area between the AFM tip and the strained FLG in the Raman maps in Fig. 2: Making the assumption that the FLG was stretching in a way so that it would conformally adhere to the AFM tip’s sidewalls for the entire depth of indentation allows us to estimate the geometrically maximum possible contact area between the tip and the FLG. This clearly sets an upper limit to the real contact area. The dimensions of our AFM tip are determined via the SEM micrographs in Supplementary Figure S2. From the force vs. indentation curves in Fig. 1(d) we estimate the maximum penetration of the tip under the maximum load (~6300 nN) to ~860 nm. For a penetration depth of ~860 nm the corresponding base of the indented section of the pyramidal AFM tip has a maximum feature size of only ~1 μm in projection onto the membrane plane (including consideration of the slanted angle of the emanating tip) i.e. as it would be observed in the Raman maps in Fig. 2. This projected feature size sets a *maximum* to the projected diameter where the FLG could potentially be in direct contact with the AFM tip (while in reality the actual contact area may be much smaller as the FLG may not touch the majority of the sidewall area of the AFM tip due to the comparably high aspect ratio of the AFM tip^41^). Given that the observed feature size of downshifted G and 2D frequencies is ~7 μm for ~6300 nN load in Fig. 2 and therefore significantly larger than the here estimated ~1 μm maximum projected contact area between AFM tip and FLG, our data clearly shows that the strain field from a nanosized SPM tip significantly extends beyond the SPM tip-membrane contact area.

**Figure 3:**
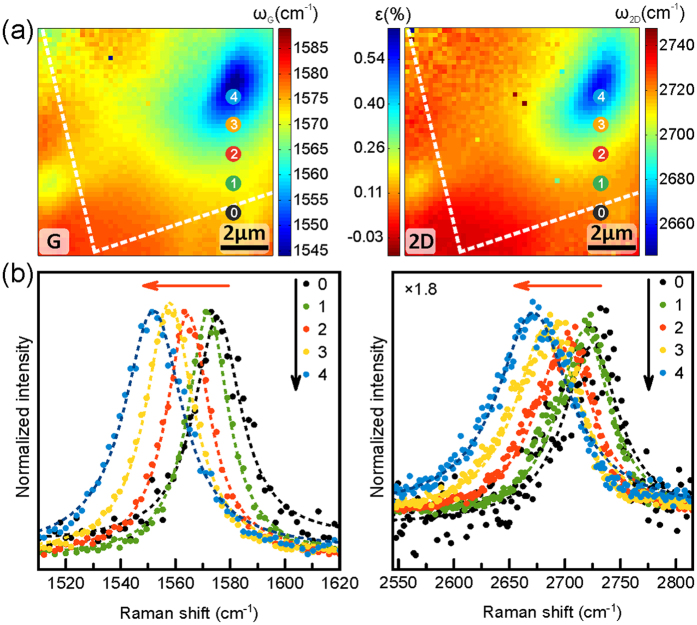
(**a**) G frequency (left) and 2D frequency/strain (right) maps for the ~6300 nN case (replotted from Fig. 2) in which the locations corresponding to the Raman spectra in (**b**) are indicated. The rim of the SiN/Si support is indicated by the white dashed line. (**b**) Raman spectra of G (left) and 2D (right) regions corresponding to the locations marked in the (**a**). Spectra were normalised to the G peak intensity and the 2D intensity panel is plotted after multiplication of intensity by ×1.8 to enhance readability.

The behaviour of the G and 2D frequencies upon the application of the maximum strain (~6300 nN) is also well reflected in the full Raman spectra at selected positions across the sample, as shown in Figs 1(e) and 3. Figure 1(e) shows individual full Raman spectra for the case of maximum applied force (~6300 nN) as a function of the numbered positions in the Si intensity map in Fig. 1(c). Within the highly G and 2D frequency downshifted region under the AFM tip we observe that the intensity of the G and 2D bands is reduced (Fig. 1(e)). This is caused by the AFM tip being placed into the laser beam path above the FLG, resulting in attenuation of the optical signal. A possible further influence on the Raman intensity could arise via the anisotropic angular distribution of Raman radiation from graphene^60^, where the slanted angle of the FLG membrane next/under to the tip under load with respect to the unloaded membrane could result in a changed overall Raman intensity. We emphasize however that for our analysis below not the Raman bands’ intensities but their frequencies are of importance. Importantly, we note that even for the region with the strongest G and 2D downshifts no defect-related D peak^47^ in the Raman spectra emerges (Fig. 1(e)). This shows that under our measurement conditions up to ~6300 nN no defects are created in the FLG lattice (i.e., no C-C bonds are broken) and that the FLG deformation is elastic. This is in agreement with the AFM force-indentation data and the observation that upon removal of the force applied by the AFM tip the Raman spectra fully reverse to their initial non-contacted states and that the FLG membrane remains visually intact.

Figure 3 shows further selected location-dependent plots of the G and 2D regions for the ~6300 nN case. Transitioning from FLG on the SiN/Si support (point “0” in Fig. 3) to under the AFM tip (“4”), we observe a gradual peak shift towards lower G and 2D frequency values. Closer inspection of Fig. 3 also reveals that not only the frequencies of the G and 2D peaks downshift but that also their peak shape evolves. In Supplementary Figures S4 and S5 we plot the widths of the peak fits corresponding to Fig. 2. We find that under and adjacent to the contacting AFM tip the 2D peak width increases from ~50 cm^−1^ for the non-loaded case to a maximum of ~90 cm^−1^ under ~6300 nN. After removal of the load, the 2D width profiles revert to their initial values. Such widening of the 2D width upon the application of stress has been reported previously for monolayer graphene^35^,^61^ and for multiple graphene layers when stress is transferred compliantly between layers^37^. The initial Raman signal of our FLG flake is consistent with AB Bernal stacked graphene layers^48^. Interlayer slippage towards turbostratic stacking in FLG has been previously shown to reduce the 2D width under strain and to lead to a drastic reduction of 2D asymmetry^37^. In contrast, we here observe a widening of the 2D width under strain and only a very small reduction in asymmetry. This suggests that under our experimental conditions no interlayer slippage in the 5-layer AB-stacked FLG towards turbostratic FLG occurs. The minor reduction in asymmetry might however point to a localised transition to ABC stacking^48^.

Beyond qualitative observations, the magnitude of the here measured G and 2D downshifts in frequency can be used to obtain a quantitative estimation of the magnitude of strain in our measurements. Commonly a linear relation between the frequency shifts of the G (∂*ω*_*G*_) and 2D bands (∂*ω*_2*D*_) and the change in strain (∂*ε*) is reported^31^,^32^,^34^. We note however that there is a significant spread in the reported literature values for the proportionality constants ∂*ω*_*G*_/∂*ε* [cm^−1^/% strain] and ∂*ω*_2*D*_/∂*ε* [cm^−1^/% strain]^34^,^38^. These are however critical to estimate the magnitude of strain from our measurements of Raman band shifts in Fig. 2. We therefore briefly discuss our considerations in this context: The largest body of data exists for monolayer graphene, for which the reported ∂*ω*_2*D*_/∂*ε* relations vary between −27 cm^−1^/% to −83 cm^−1^/% for uniaxial strain^31^,^32^,^34^, −144 cm^−1^/% to −203 cm^−1^/% for biaxial strain^32^,^34^,^40^ and −140 cm^−1^/% for a radial geometry^36^. Similar spread is also found in the reports of ∂*ω*_*G*_/∂*ε* for monolayer graphene, where additionally peak splitting into G^−^ and G^+^ has to be considered, which is absent for FLG^32^,^34^. Similarly for FLG and graphite the reported proportionality constants have a significant spread: For 3-layer FLG under uniaxial strain ∂*ω*_2*D*_/∂*ε* of −22 cm^−1^/% and ∂*ω*_*G*_/∂*ε* cm^−1^/% of −12 cm^−1^/% were reported^31^, while for graphite ∂*ω*_2*D*_/∂*ε* of −154 cm^−1^/% for biaxial strain was indicated^32^. In the measurements in Fig. 2, a FLG flake of 5 layers was measured (placing it somewhere between 3-layer FLG and graphite) and the strain was induced by the highly localised load from an indenting tip, resulting in a location dependent radial and circumferential strain type^43^. This makes it non-trivial to select the most appropriate proportionality constants from literature. We therefore adopt for an *estimation* of strain a ∂*ω*_2*D*_/∂*ε* of −140 cm^−1^/% from ref. 36 (radial geometry for monolayer graphene) which is also numerically very close to the value for biaxial strain for graphite (−154 cm^−1^/%) from ref. 32. We note however that the thus derived magnitudes of strain critically hinge on the selection of this proportionality constant. For our maximum 2D downshifts in Fig. 2(e–h) we thus estimate maximum strain levels of ~0.15% for ~1300 nN, ~0.20% for ~3800 nN, and ~0.56% for ~6300 nN, respectively. Additionally the thus calculated spatially resolved strain levels are plotted as a scalebar alongside Fig. 2(e–h). In this context, we however emphasize that our here reported novel methodology is not aimed at definitely measuring the absolute magnitude of strain, but instead at clarifying the *relative spatial extent* of the localised strain distribution which is unaffected by the selection of the proportionality constants.

**Figure 4:**
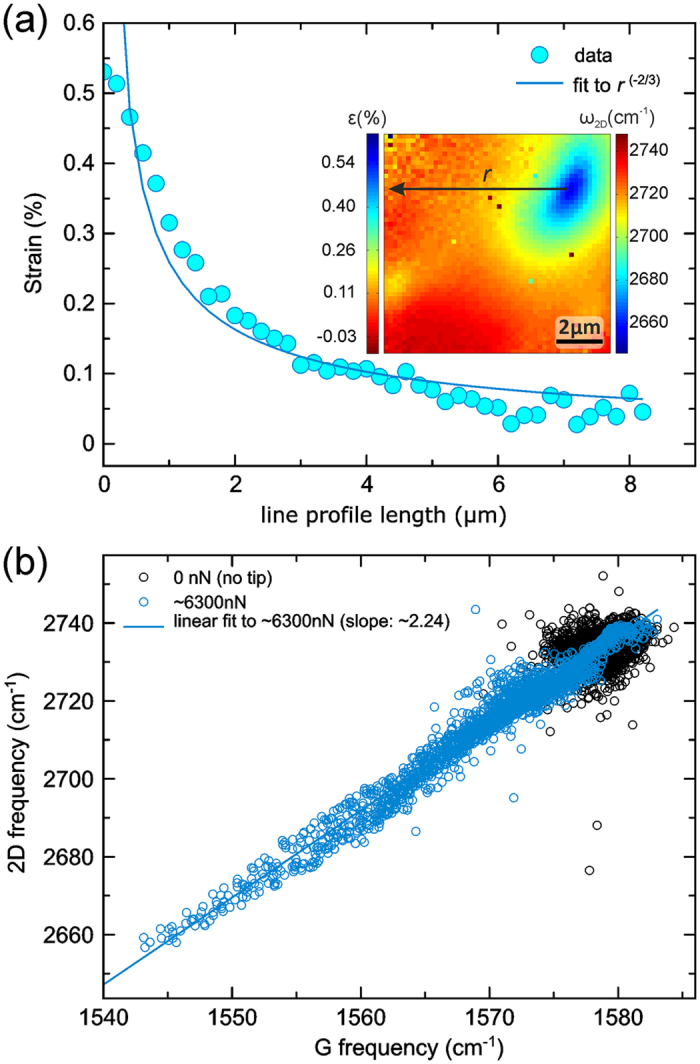
(**a**) Line profile of experimentally determined strain values (as described above) for ~6300 nN as a function of position along the black line “r” marked on the 2D frequency/strain map in the inset (map replotted from Fig. 2). The fit to the experimental data follows *ε*_*total*_ ~*C* × *r*^(−2/3)^ with *r* being the radial distance from the point of indentation and *C* as the free fitting parameter^43^. Recalculation^43^ of *C* yields an indentation depth from the fit on the order of ~400 nm, largely consistent with the measured ~600 nm indentation depth (if the initial flattening of wrinkling for the initial ~250 nm indentation is taken into account). (**b**) Correlation plots of 2D and G Raman peak frequencies for the non-loaded 0 nN and the ~6300 nN cases. For the ~6300 nN case, a linear fit to the data reveals a slope Δ*ω*_2*D*_/Δ*ω*_*G*_ of 2.24 ± 0.03 45.

To assess the validity of our strain distribution measurements, we compare our experimental findings with the results from an analytical model for the radial and circumferential strain distribution in a membrane from a point deformation. As discussed in ref. 43 the total strain *ε*_*total*_ from a point deformation to a membrane is the sum of radial *ε*_*radial*_ and circumferential *ε*_*circum*_ components. The total strain *ε*_*total*_ has a radial dependence with radial distance *r* from the point of indentation of *ε*_*total*_ ~*r*^(−2/3)^. Figure 4(a) shows an extracted line profile through the AFM tip contact location from the 2D frequency/strain map for ~6300 nN in the inset. We find that this line profile is well fitted with such a *r*^(−2/3)^-dependence (up to the estimated radius of the AFM tip where the measured strain plateaus, while the fit tends towards infinity). Extracted^43^ indentation depth from this fit (~400 nm) is in reasonable agreement with the measured value (~600 nm, when the initial flattening of FLG wrinkles within the first ~250 nm indentation is taken into account). This indicates good agreement between our measurements and the analytical model (in particular given that the experimental strain estimation critically hinges on the selection of ∂*ω*_2*D*_/∂*ε*) and thus validates that our combined AFM-Raman approach can also be used to measure membrane strain in a quantitative manner.

One possible complication to our Raman spectroscopy-AFM based methodology needing consideration is laser induced heating of the FLG during our measurements^62^,^63^. Such laser induced heating could potentially also lead to downshifts in the FLG Raman signature due to thermal expansion^62^,^64^,^65^. Comparison to literature however suggests that direct heating of the FLG via absorption of the laser light is negligible for the wavelength and power used: In particular, for the here used 3.5 mW at 473 nm a G peak frequency downshift of only around −4 cm^−1^ would be expected from thermal effects^62^, far smaller than our here observed G frequency downshifts upon load of up to −38 cm^−1^. Another possibility requiring consideration is laser absorption in the AFM tip resulting in laser induced localised heating of the AFM tip and thus indirect heating of the FLG when in contact with the AFM tip. We note however that the frequencies of the first order Si Raman signal at the location of the AFM tip compared to at the location of the macroscopic SiN/Si support show negligible variation. Given that macroscopic supports under our Raman conditions show negligible heating^62^ and given the temperature sensitivity of the Si Raman bands^52^, this excludes significant laser induced heating of the AFM tip. Based on these arguments, thermal effects can safely be considered to be negligible for the here presented measurement conditions.

Another possible complication to our analysis of AFM tip load induced strain distribution via Raman peak shifts arises from the dependence of graphene Raman peak shifts also on charge transfer doping^45^. When in contact with the FLG, the Pt-coated Si AFM tip could lead to a charge transfer between the Pt and the FLG (in essence the FLG is then “supported” by the AFM tip from the top). This could result in Raman peak shifts from transfer doping in addition to the Raman shift from strain. While our choice of FLG as a model system is partly motivated by the reduced charge transfer effects in FLG (via screening between layers) compared to monolayer graphene^66^, in the following paragraph we disentangle strain and possible charge transfer effects^45^.

From tapping mode AFM imaging of the FLG membrane in Supplementary Figure S1, we observe that the exfoliated FLG flake is partly covered by small amounts of polymeric residues from the dissolved plastic TEM grid used for intermediate FLG placement^14^,^49^. The largely featureless G and 2D frequency maps for the non-loaded case (0 nN, i.e., no AFM tip; Fig. 2(a,e)) reveal that any doping possibly resulting from the residues^67^ is homogeneous across the sample within the spatial resolution of the Raman mapping. In our estimation of AFM tip-membrane contact area above we found for maximum load (~6300 nN) a maximum projected tip-membrane contact area diameter of ~1 μm. This sets the maximum projected feature size where direct charge transfer could occur to ~1 μm, which is significantly smaller than the ~7 μm size of the observed area of the strong Raman peaks shifts in Fig. 2(d,h). Therefore charge transfer is not an issue for the majority of this area being not in direct contact with the AFM tip. To estimate the possibility of charge transfer in the remaining central region of direct contact to the Pt-coated tip, we consider that graphene in direct contact with Pt is known to become p-type doped^68^. Both p-type and n-type doping of graphene result in an *upshift* of the G band^45^,^69^. For Pt nanoparticles directly on graphene an upshift of +3 cm^−1^ was reported^70^. Contrary to this, we observe a strongly *downshifted* G band also in central area of tip contact (Fig. 2(d)). This suggests that even in the central contact area any possible charge transfer between the FLG and the Pt-coated AFM tip is negligible compared to the strain induced from the mechanical deformation.

The conclusion that strain is the dominating mechanism behind the evolution of the Raman features in Figs 2 and 3 is finally further corroborated when we follow the analysis methodology of ref. 45 by plotting in Fig. 4(b) the correlation between 2D and G frequencies for the 0 nN and ~6300 nN cases. For the non-loaded 0 nN case we find a distribution of 2D and G values, which results from the intrinsic wrinkling of the FLG membrane as well as instrumental scatter^42^,^45^. When then contacted by the AFM tip (~6300 nN), this distribution stretches towards downshifted 2D and G frequencies, where the most downshifted data points correspond to the FLG region directly under the AFM tip (see Supplementary Figure S6 for a visualisation of the location-dependence of this 2D-G correlation). Importantly, the slope Δ*ω*_2*D*_/Δ*ω*_*G*_ of this ~6300 nN distribution has a value of 2.24 ± 0.03, which is in stark contrast to the slope value expected for a doping-driven Raman frequency shift (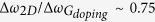), but is in excellent agreement with the slope value expected for a purely strain driven Raman frequency shift (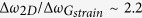)^45^. This further affirms that our concurrent AFM-Raman method successfully reveals the strain evolution in FLG membranes under localised deformation in the elastic regime.

## Conclusions

In summary, we have demonstrated, using FLG as model system, that the combination of simultaneous AFM measurements and hyperspectral Raman mapping allows unprecedented insights into the spatial distribution of strain from point deformations in two-dimensional materials. In the presented experiments we have used an AFM tip as a nanoindentation probe to controllably induce highly localised strain in free-standing FLG in the elastic regime, where we visualise its strain distribution using independently controlled laterally resolved Raman spectroscopy via measuring strain-dependent frequency shifts of the FLG’s G and 2D Raman bands. Our approach is extendible towards other SPM-type actuation modes and force regimes as well as towards many other two-dimensional materials that can be optically probed. Thus the presented methodology contributes to the instrumental toolbox for controlled and highly localised strain engineering in suspended two-dimensional materials, crucial to a wide variety of further fundamental mechanical studies and envisaged applications.

## Methods

Suspended FLG is prepared over single 12 μm × 12 μm holes in holey SiN-covered Si chips (SiMPore) by mechanical exfoliation from HOPG. HOPG is first exfoliated onto SiO_2_ covered Si wafers and then transferred onto NMP dissolvable plastic TEM grids (Quantifoil) as intermediate carriers to deterministically transfer the FLG flakes onto the holey SiN/Si chips^14^. The FLG flakes are characterized by optical contrast difference on the initial SiO_2_/Si substrate^46^ and Raman spectroscopy^47^,^48^. A NT-MDT NTEGRA Spectra coupled AFM-Raman spectrometer is used in our experiments. In this setup the sample is located on a *x,y,z*-moveable piezo tube scanner under the fixed AFM probe, which has the tip emanating in front of the cantilever at an angle of ~128° to allow visual access to the sample-tip contact (silicon AFM probes coated with Pt, NT-MDT VIT_P_Pt). Independent of the AFM control, the Raman laser spot (473 nm) is laterally *x,y*-moveable on the sample surface via a mirror mounted onto a second piezo tube in the laser beam path. This allows to perform AFM measurements (such as nanoindentation) on an area of interest while simultaneously and independently rastering the Raman laser spot over the same area of interest to obtain hyperspectral Raman maps *in situ* during the AFM measurements.

## Additional Information

**How to cite this article**: Elibol, K. *et al*. Visualising the strain distribution in suspended two-dimensional materials under local deformation. *Sci. Rep.* **6**, 28485; doi: 10.1038/srep28485 (2016).

## Supplementary Material

Supplementary Information
