# Supplementary material for: Visualising the strain distribution in suspended two-dimensional materials under local deformation
Source: Sci Rep. 2016 Jun 27;6:28485. doi: 10.1038/srep28485 (PMC4921963; doi:10.1038/srep28485)
Supplement: Supplementary Information [file srep28485-s1.pdf]

SUPPLEMENTARY INFORMATION

Visualising the strain distribution in suspended two-dimensional materials under local deformation

Kenan Elibol,[1] Bernhard C. Bayer,[1,*] Stefan Hummel,[1] Jani Kotakoski,[1] Giacomo Argentero,[1] Jannik C. Meyer[1]

[1] *Faculty of Physics, University of Vienna, Boltzmanngasse 5, A-1090 Vienna, Austria*

[*] Corresponding author: email: bernhard.bayer@univie.ac.at, tel.: +43-1-4277-72870

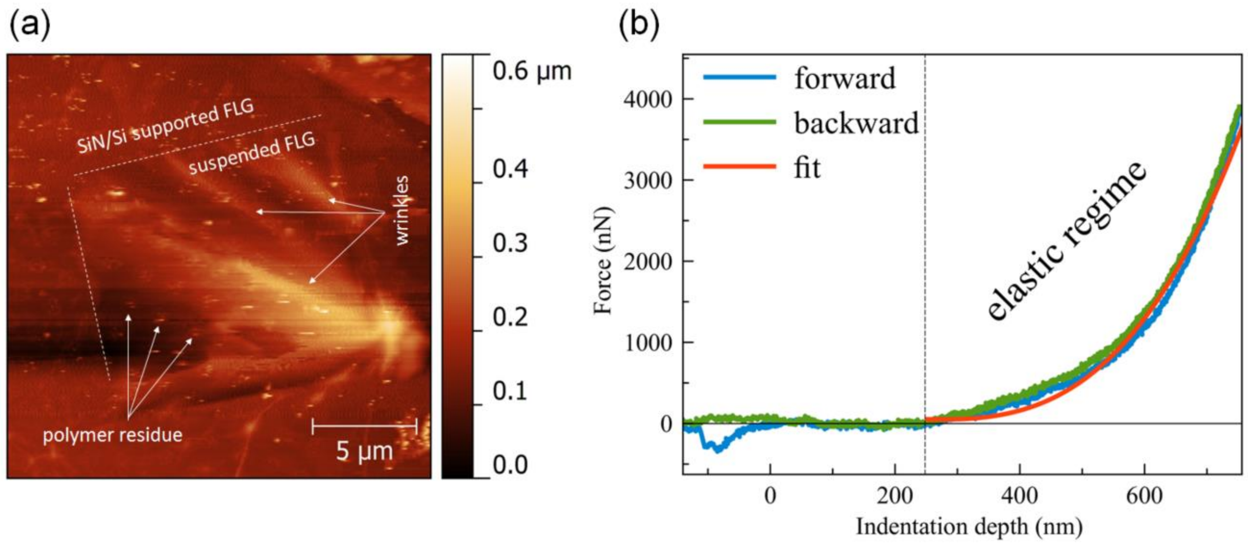

Supplementary Figure S1: (a) Tapping mode atomic force microscope (AFM) topography image of the few-layer graphene (FLG) membrane with its salient features indicated. (b) Forward and backward trace of the force-indentation curve from Fig. 1(d) as well as a fit to the experimental forward trace data following Lee et al. *Science* **321**, 385–388 (2008).

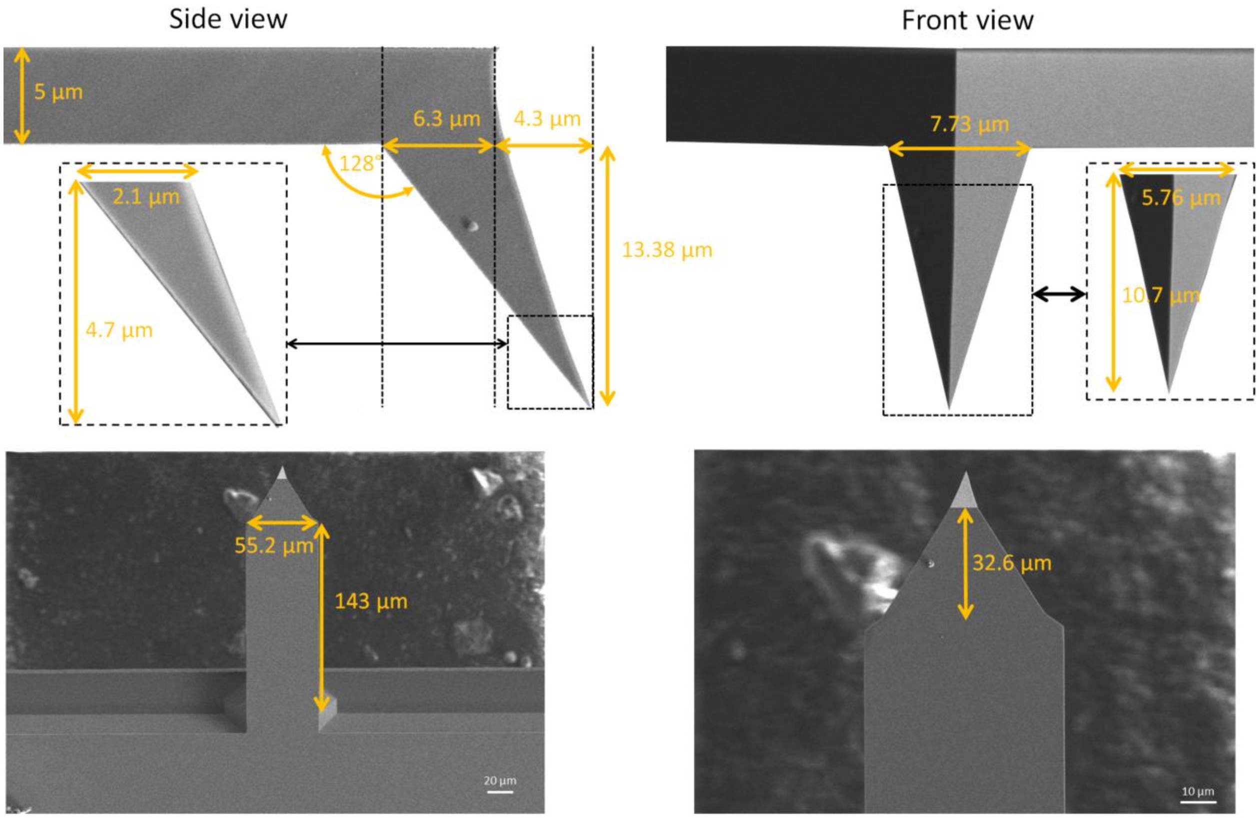

Supplementary Figure S2: Scanning electron micrographs to measure the dimensions of the NT-MDT VIT_P_Pt AFM probe.

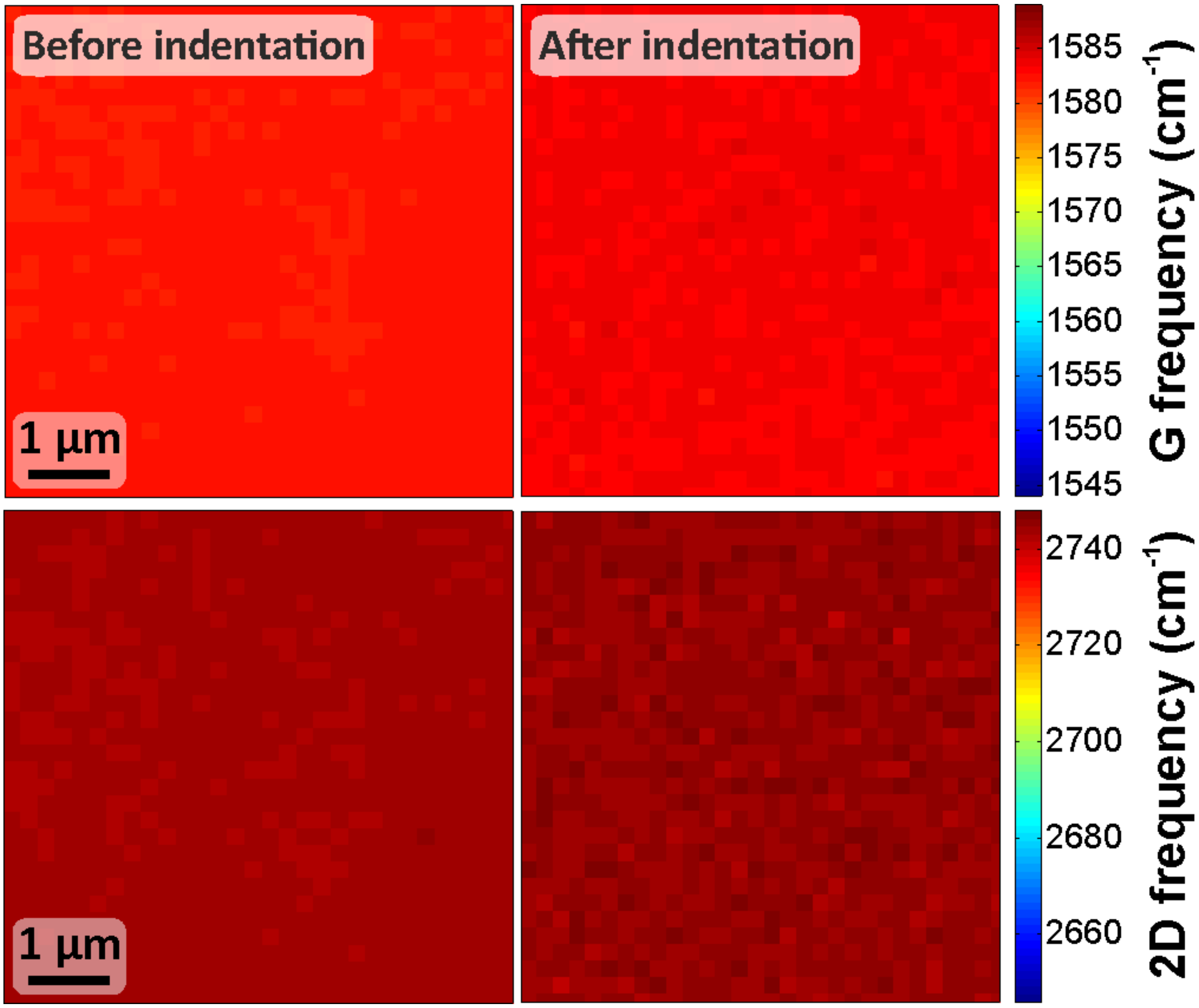

Supplementary Figure S3: G and 2D frequencies measured without an AFM tip in place on a 7 layer FLG membrane before and after indentation at similar AFM tip loads as in Fig. 2. The data shows that the membrane remains intact under indentation and that Raman features (such as in Fig. 2) are reversible. (Note that this membrane is another sample than the 5 layer membrane with which the remaining exemplary data in the paper was measured.)

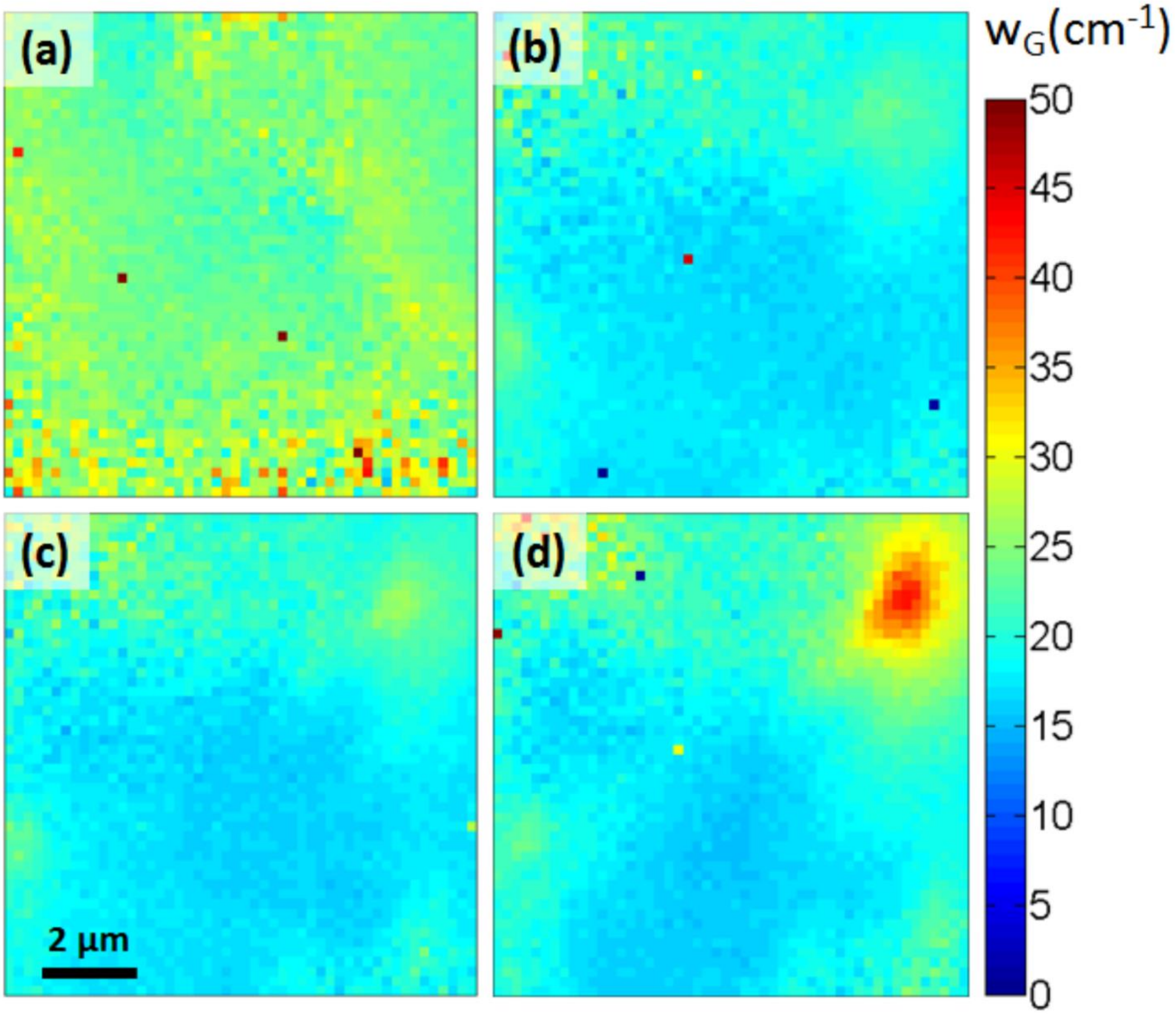

Supplementary Figure S4: Raman maps of the widths (w_G) of the G peaks corresponding to Fig. 2(a-d). Forces applied by AFM probe are (a) 0 nN (no tip in place), (b) ~1300 nN, (c) ~3800 nN, and (d) ~6300 nN, respectively.

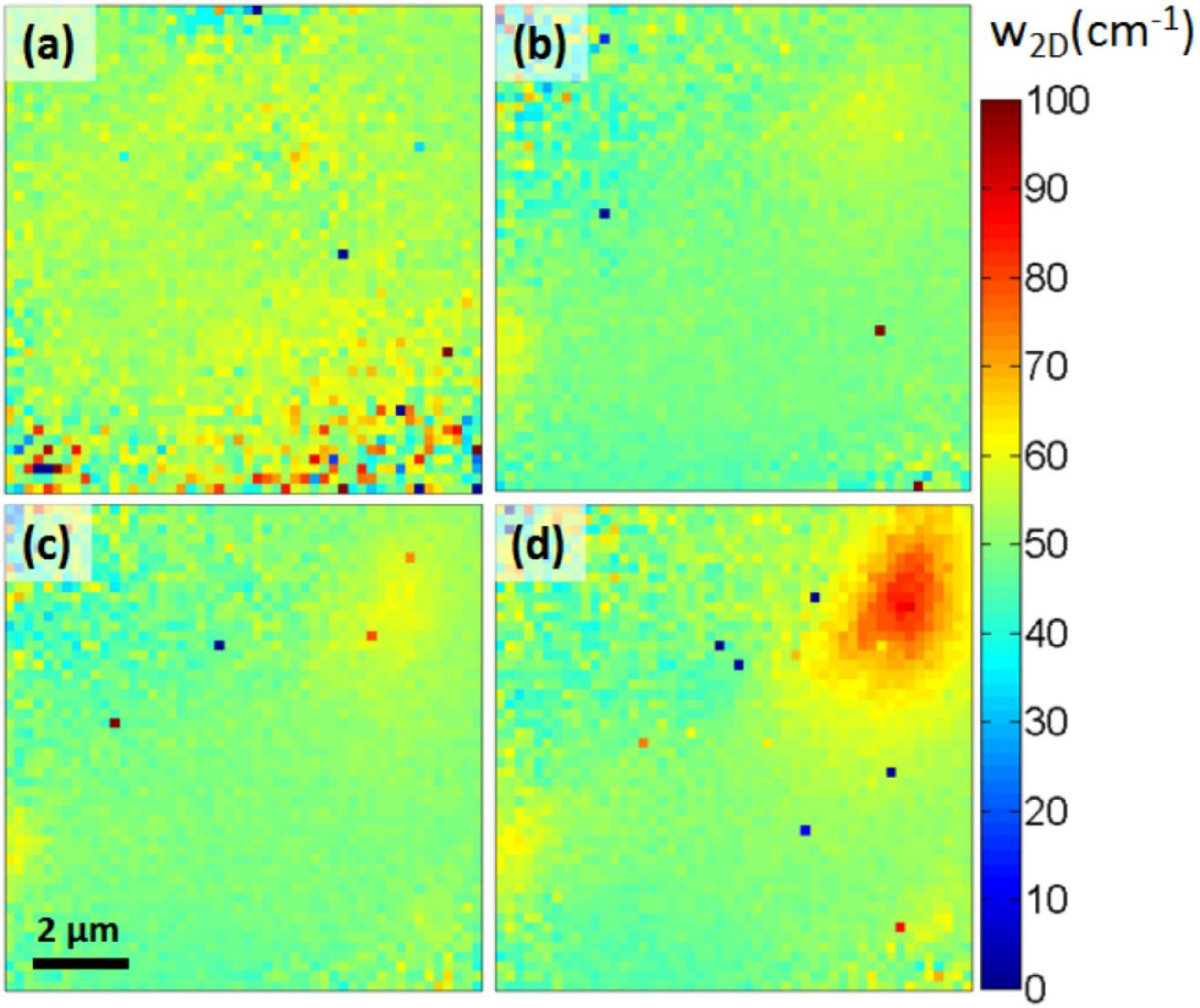

Supplementary Figure S5: Raman maps of the widths (w_{2D}) of the 2D peaks corresponding to Fig. 2(e-h). Forces applied by AFM probe are (a) 0 nN (no tip in place), (b) ~1300 nN, (c) ~3800 nN, and (d) ~6300 nN, respectively.

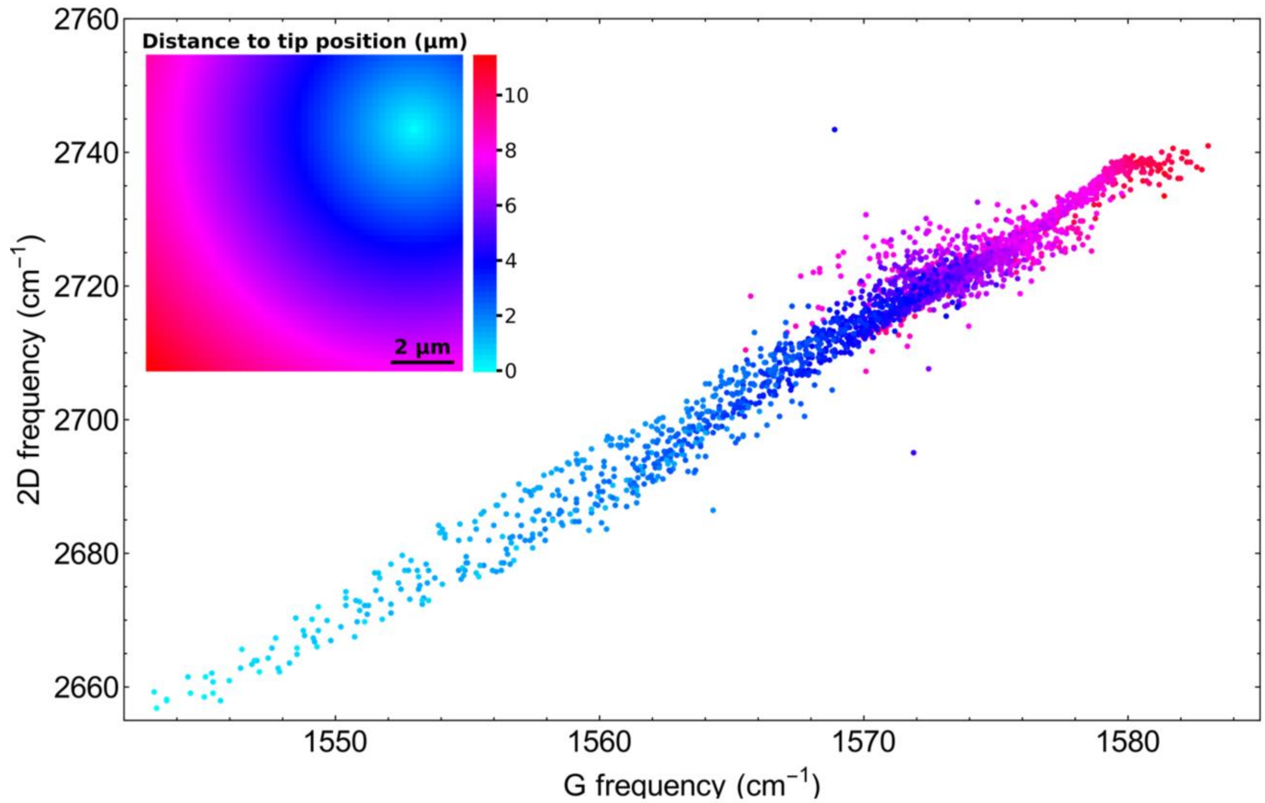

Supplementary Figure S6: Location-resolved correlation plot of 2D and G Raman peak frequencies for the ~6300 nN case. The colour of the data points encodes the distance from the AFM tip position, proving that the largest downshifts for both peaks occur under the tip. Colour map showing the distance to the tip position is shown in the inset.
